# Effects of Compositional Ratio of Ti-Al-C on Formation of Ti_2_AlC by Self-Sustaining Combustion Synthesis

**DOI:** 10.3390/ma19061100

**Published:** 2026-03-12

**Authors:** Chun-Liang Yeh, Yu-Ting Chen

**Affiliations:** Department of Aerospace and Systems Engineering, Feng Chia University, Taichung 40724, Taiwan

**Keywords:** Ti_2_AlC, combustion synthesis, deficient carbon, excess Al, weight fraction

## Abstract

The formation of Ti_2_AlC was investigated by self-propagating high-temperature synthesis (SHS) from the elemental Ti-Al-C powder compacts. The compositional ratios of Ti:Al:C varied from 2:1:1 to 2:1.2:0.8 to explore the effects of deficient carbon and excess Al on the combustion kinetics and product formation. For the Ti-Al-C powder compacts, self-sustaining combustion featuring a distinct combustion wave was readily achieved upon ignition. Excess Al caused a decrease in combustion temperature and flame-front velocity, while deficient carbon showed relatively little influence. The synthesized product from the sample with an exact stoichiometry of Ti:Al:C = 2:1:1 was composed of 79.5 wt.% Ti_2_AlC, 9.8 wt. Ti_3_AlC_2_, 10.7 wt.% TiC, and a small amount of Ti_3_AlC. The addition of excess Al by 20 at.% not only increased the yield of Ti_2_AlC but avoided the formation of Ti_3_AlC. A reduction of carbon further improved the evolution of Ti_2_AlC. The sample with an off-stoichiometric proportion of Ti:Al:C = 2:1.2:0.9 yielded the optimum product composition of 91.9 wt.% Ti_2_AlC, 4.2 wt.% Ti_3_AlC_2_, and 3.9 wt.% TiC. This was attributed to the fact that excess Al and deficient carbon facilitated the formation of TiAl and sub-stoichiometric TiC, both of which acted as the intermediate phases to combine into Ti_2_AlC. The as-synthesized Ti_2_AlC grains were in the shape of thin platelets with a size of 4–8 μm and a thickness of about 1.0 μm. A laminated microstructure formed by closely stacked platelets is typical of the MAX carbide.

## 1. Introduction

MAX phases are a group of layered hexagonal carbides and nitrides and can be designated by a formula of M*_n_*_+1_AX*_n_* with *n* = 1, 2, and 3, where M corresponds to an early transition metal, A to an A-group element (mainly IIIA and IVA), and X to either C or N [1]. Depending on the value of index *n*, MAX phases are generally divided into three categories, namely 211, 312, and 413 groups. These materials are of interest because they feature both metallic and ceramic properties. Like metals, they exhibit excellent electrical and thermal conductivity, good machinability, outstanding thermal shock resistance, and plasticity at high temperatures. Their ceramic-like characteristics include low density, good oxidation resistance, low thermal expansion coefficient, high melting point, high modulus and strength, and good thermal stability [1,2,3,4].

The Ti-Al-C system contains three carbides, i.e., Ti_2_AlC, Ti_3_AlC, and Ti_3_AlC_2_, with melting points of 1625, 1580, and 1360 °C, respectively [5]. Ti_2_AlC has the highest melting point. Moreover, the low density of 4.11 g/cm^3^ positions Ti_2_AlC as the lightest Ti-based 211 MAX phase. Hardness, electrical conductivity, and thermal conductivity of Ti_2_AlC are 4–5 GPa, (2.7–4.5) × 10^6^ Ω^−1^·m^−1^, and 46 W/m·K, respectively [6]. Compressive strength, bending strength, and fracture toughness of Ti_2_AlC are 670 MPa, 384 MPa, and 7 MPa·m^1/2^, respectively [7]. Due to its excellent resistance to oxidation at elevated temperatures and mechanical properties, Ti_2_AlC has been considered as the most practical MAX phase for high-temperature structural applications in the form of bulk and coatings [8,9]. The outstanding oxidation resistance of Ti_2_AlC is attributed to the formation of an α-Al_2_O_3_ layer on the surface at 1000 °C. Especially, close values of the thermal expansion coefficient of Al_2_O_3_ and Ti_2_AlC make it not easy to separate from the substrate and reduce the thermal stress at high temperatures [8,9]. Ti_2_AlC also demonstrates superior crack self-healing capabilities. It was found that superficial cracks and grooves of a width ≤ 10 μm on Ti_2_AlC were self-healed after 20 h oxidation at 1200 °C via dense Al_2_O_3_ [10]. The self-healing temperature of Ti_2_AlC coatings was further reduced to below 700 °C by A-site Sn solid solution [11]. Moreover, the defects generated during corrosion could be effectively self-healed by the Al_2_O_3_ amorphous phase, thus enhancing the corrosion-resistant properties of Ti_2_AlC coatings [12,13].

Practical applications of Ti_2_AlC and other MAX phases like Ti_3_AlC_2_ and Cr_2_AlC include high-temperature coatings for gas turbines, nuclear fuel cladding, electrical contacts, energy storage devices, and catalyst supports [14,15,16,17,18]. The use of Ti_2_AlC in aerospace and industrial gas turbines is attributed to its excellent oxidation resistance and thermal expansion compatibility with protective ceramic layers [14,15]. Ti_2_AlC and Ti_3_AlC_2_ are potential candidates for accident-tolerant fuel cladding in nuclear reactors because they are resistant to irradiation and corrosion in high-temperature environments [16,17]. Ti_2_AlC and Ti_3_AlC_2_ are suitable for electrical contacts, resistors, and conducting materials due to their high electrical conductivity [14,15,16,17]. Ti_2_AlC has been primarily used as a source material for etching to produce 2D MXene materials for advanced electronics and energy storage [15,18].

Synthesis routes to produce Ti_2_AlC have been diverse, and different reactant mixtures and compositional ratios have been adopted. The methods include hot pressing (HP) [19], hot isostatic pressing (HIP) [6], solid–liquid reaction synthesis [20], spark plasma sintering (SPS) [21,22], pressureless sintering [23,24], microwave sintering [25], molten salt synthesis (MS) [9,26], self-propagating high-temperature synthesis (SHS) [27,28,29,30,31], and mechanical-activated thermal explosion [32,33]. Barsoum et al. [6,19] produced bulk polycrystalline Ti_2_AlC from a Ti-Al_4_C_3_-C powder mixture by HIP for 15 h at 1300 °C and 40 MPa and HP for 4 h at 1600 °C and 40 MPa. Wang and Zhou [20] developed a solid–liquid reaction process operating at 1400 °C and 30 MPa for 1 h to fabricate Ti_2_AlC from a stoichiometric elemental powder compact. By using the SPS technique, Zhou et al. [21] obtained dense Ti_2_AlC from an Al-excessive powder mixture with a molar ratio of Ti:Al:C = 2:1.2:1 at 1100 °C and 30 MPa for 1 h. With Ti, TiC, and Al_4_C_3_ as the precursor materials at a stoichiometry of Ti:TiC:Al_4_C_3_ = 7:1:1.3 (i.e., a 30 mol.% excess of Al_4_C_3_), Ti_2_AlC of 90 wt.% purity was synthesized by SPS at a sintering temperature of 1300 °C [22]. Benitez et al. [23] utilized Ti, Al, and TiC as the reactants with a molar ratio of Ti:Al:TiC = 1.00:1.05:0.95 to prepare Ti_2_AlC by pressureless sintering in a tube furnace at 1400 °C under argon. Gauthier-Brunet et al. [24] produced Ti_2_AlC from a powder mixture composed of Ti:C:Al_4_C_3_ = 8:1:1 by reactive sintering. Three intermediate phases, including TiC, Ti_3_Al, Ti_3_AlC, were detected. After sintering at 1300 °C and 1400 °C, the dominant phases were Ti_2_AlC and TiC [24]. Microwave sintering, a distinctive manufacturing route, offering rapid volumetric heating and effective interfacial interactions, was employed to prepare Ti_2_AlC from stoichiometric Ti, Al, and TiC powders [25]. By means of the MS method, Badie et al. [9] adopted elemental Ti, Al, and graphite powders with different Ti:Al:C stoichiometries of 2:1:1, 2:1.1:1, 2:1.05:1, 2:1:0.8, 2:1:0.9, and 2:1:0.95 to mix with KBr for the synthesis of Ti_2_AlC at 950, 1000, and 1050 °C for 1, 5, 10, and 15 h. Results showed that a powder mixture with a carbon-deficient composition of 2:1:0.9 produced the highest yield of Ti_2_AlC up to 91 wt.%. In addition to Ti_2_AlC, many secondary phases, such as Ti_3_AlC_2_, TiC, TiAl, Ti_3_Al, and Ti, were present in end products from other reactant mixtures [9]. Using a similar MS technique, Nadimi et al. [26] obtained Ti_2_AlC at 1100 °C for 1.5 h from reactants made up of elemental Ti, Al, and graphite powders with a molar ratio of 2:1:1 and a mixture of NaCl and KCl salts.

Another promising method for preparing the MAX phases, like Ti_2_AlC, Ti_3_SiC_2_, and Ti_3_AlC_2_, is combustion synthesis in the SHS mode, where the exothermic reaction is exploited and many merits are recognized, including energy efficiency, short reaction time, simplicity, and cost effectiveness [34,35]. According to Hashimoto et al. [27], Ti_2_AlC was fabricated by the SHS technique from a compacted powder mixture consisting of Ti:Al:C = 2:1:1, and the final product was Ti_2_AlC along with a small amount of TiC_0.57_ due to the loss of Al during the SHS process. Thomas and Bowen [28,29] studied the effect of the Al amount on the synthesis of Ti_2_AlC from elemental powder mixtures by SHS and indicated that excess Al of 10–30 at.% reduced the combustion temperature and slowed down the cooling of the sample upon completion of the reaction, thus favoring the formation of Ti_2_AlC. Effects of TiC and Al_4_C_3_ as reactant materials on the synthesis of Ti_2_AlC by SHS were studied using Ti/Al/C/TiC and Ti/Al/C/Al_4_C_3_ powder mixtures, both of which possessed a stoichiometric ratio of Ti:Al:C = 2:1:1 [30]. The addition of TiC facilitated the formation mechanism and enhanced the degree of Ti_2_AlC evolution to around 90 wt.%. However, Al_4_C_3_-added samples reduced the reaction exothermicity and led to a slight decrease in the yield of Ti_2_AlC [30]. Aydinyan [31] added PTFE of 2 wt.% as a reaction activator into a mixture of 2Ti/1.4Al/0.9C to synthesize Ti_2_AlC via an activated SHS process. PTFE promoted the reaction and phase conversion. The final product was composed of Ti_2_AlC and TiC at a weight ratio of 92.2:7.8 [31].

Thermal explosion (TE) is another mode of combustion synthesis and involves uniform heating throughout the entire sample, leading to rapid bulk reactions [36]. Khoptiar et al. [32] synthesized Ti_2_AlC of 90 wt.% purity from elemental Ti, Al, and C powders via mechanical activation for 3 h, followed by TE with preheating at 800 °C and compressing at 30 MPa. Edrisi et al. [33] conducted TE synthesis to fabricate Ti_2_AlC with mechanically activated powder mixtures of Ti:Al:C = 2:1:1 at a preheating temperature of 1000 °C. Mechanical activation lowered the formation temperature of Ti_2_AlC and reduced the secondary phase, thereby producing a synthesized product containing Ti_2_AlC of 96.1 wt.% [33].

According to the literature reviewed above, initial powder mixtures in off-stoichiometric proportions, i.e., Al-excessive and carbon-deficient compositions, had a positive effect on the formation of Ti_2_AlC. However, such effects caused by Al excess and carbon reduction on the synthesis of Ti_2_AlC have not been fully studied by SHS. This study intended to investigate the production of Ti_2_AlC from elemental powder mixtures by solid state combustion in the SHS mode. The initial stoichiometric ratios of Ti:Al:C varied from 2:1:1 to 2:1.2:0.8 to examine the influence of Al and carbon contents on the phase conversion of Ti_2_AlC. In this work, the self-sustaining combustion behavior was assessed, and measurement of the combustion wave velocity and reaction temperature was conducted. Analysis of the microstructure and constituent composition of SHS-derived products was performed. Furthermore, the potential reaction steps to form Ti_2_AlC via the SHS route were proposed.

## 2. Materials and Methods

In this study, the raw materials included Ti (Alfa Aesar, Ward Hill, MA, USA, <45 μm, and 99.8%), Al (Alfa Aesar, Ward Hill, MA, USA, <45 μm, and 99.7%), and carbon black (Showa Chemical Co., Tokyo, Japan). The composition of the test sample was expressed as Equation (1), where the stoichiometric parameters, *x* and *y*, signify the number of moles of Al and carbon, respectively, in the mixture of reactant powders. It should be noted that Equation (1) represents a nominal compositional design rather than a balanced chemical reaction. Values of *x* equal to 1.0, 1.1, and 1.2 were adopted to investigate the effect of excess Al, i.e., an Al-rich composition. The amount of carbon was studied by considering values of *y* at 0.8, 0.9, and 1.0 to assess the influence of carbon reduction, i.e., a carbon-lean test condition. In total, green samples with nine initial compositions were prepared.

(1) $$2Ti+xAl+yC\to {Ti}_{2}AlC$$

The reactant powders were dry-mixed in a tumbler ball mill. Teflon milling jars were used and were partly filled with raw materials and alumina grinding balls, and the cylindrical jar rotated about the longitudinal axis of the ball mill. Non-sticky Teflon jars have been generally utilized owing to their minimum contamination and easy-to-clean benefits. Alumina grinding balls had a diameter of 1 mm, and the ball-to-powder ratio was 7:1. The speed of the tumbler mill machine was 75 rpm, and the milling time was 4 h. Then, the powder mixture was compressed uniaxially into sample compacts of a cylindrical shape with a diameter of 7 mm, a height of 12 mm, and a relative density of 50%. The Ti-Al-C sample of 50% relative density was easy to compress, and the rigidity of the powder compact was suitable for handling in the experiment. The relative density of 50% for the test specimen was related to the initial elemental mixture. The theoretical density (*ρ*_TD_) of the test specimen was calculated from the mass fraction (*Y*) and density (*ρ*) of each component based on Equation (2).

(2) $$\frac{1}{\rho_{TD}}=\frac{Y_{Ti}}{\rho_{Ti}}+\frac{Y_{Al}}{\rho_{Al}}+\frac{Y_{C}}{\rho_{C}}$$

The SHS experiment was performed in a windowed stainless-steel combustion chamber filled with Ar at 0.25 MPa. A heated tungsten coil was utilized as the igniter to initiate the combustion reaction from the top surface of the power compact. From the recorded time-sequence film images, the propagation velocity of the combustion wave (V_f_) was deduced. The combustion temperature of the sample was measured by a bare-wire thermocouple (Pt/Pt-13%Rh). The wire diameter of the thermocouple was 62.5 μm, junction bead size 125 μm, and leg length 40 mm. The thermocouple bead was attached firmly to the surface of the sample at a location about 7 mm below the ignition top plane. This location for combustion temperature measurement was justified by the assumption that self-sustaining combustion would be well developed at this position. The measurement accuracy of a fine-wire thermocouple is influenced by conduction cooling and radiation loss. The heat loss of the thermocouple through conduction depends on the length of wire between the junction and the support. The leg length of the thermocouple used in this study ensured that the measurement would be reasonably unaffected by the conduction cooling. The radiation heat loss from the bead junction is another source of error in the thermocouple measurement. An estimation of the radiation correction was performed by considering a steady state between convective heat transfer and radiation loss from the thermocouple bead junction. The radiation correction for the temperature range of this study was about 15–25 °C, which was more pronounced at higher temperatures. It is believed that the accuracy of the combustion temperature measurement was within ±5 °C after the radiation correction. Details of the experimental methodology have been published elsewhere [37].

The constituent composition of the SHS-derived product was analyzed by an X-ray diffractometer (XRD, Bruker D2 Phaser, Karlsruhe, Germany) with CuK_α_ radiation and wavelength λ = 1.5406 Å. The operating voltage was 40 kV, and the current was 30 mA. The step size was 0.05°, and the scanning speed was 2°/min. The scan range spanned from 5° to 80° (2θ). The microstructure and elemental composition of the final product were examined with a scanning electron microscope (SEM) and an energy dispersive spectrometer (EDS) (Hitachi, S3000H, Tokyo, Japan). A reference intensity ratio method based on the XRD spectrum was utilized to quantitatively characterize the phase composition [38]. Three XRD diffraction peaks were selected to calculate the weight fractions of Ti_3_AlC_2_, Ti_2_AlC, and TiC in the synthesized product according to Equations (3)–(5) [38]:

(3) $$W_{a}=\frac{I_{a}}{I_{a}+0.220I_{b}+0.084I_{c}}$$

(4) $$W_{b}=\frac{I_{b}}{4.545I_{a}+I_{b}+0.382I_{c}}$$

(5) $$W_{c}=\frac{I_{c}}{11.905I_{a}+2.619I_{b}+I_{c}}$$

where *W_a_*, *W_b_*, and *W_c_* are the weight percentages of Ti_3_AlC_2_, Ti_2_AlC, and TiC, respectively. *I_a_*, *I_b_*, and *I_c_* signify the integrated diffraction peak intensities of Ti_3_AlC_2_ (002) at 2θ = 9.5°, Ti_2_AlC (002) at 2θ = 13.0°, and TiC (111) at 2θ = 35.9°; these three signature peaks were not overlapped with those of other phases and were selected to perform the quantitative phase analysis [38].

## 3. Results

### 3.1. Combustion Wave Velocity and Combustion Temperature

**Figure 1:**
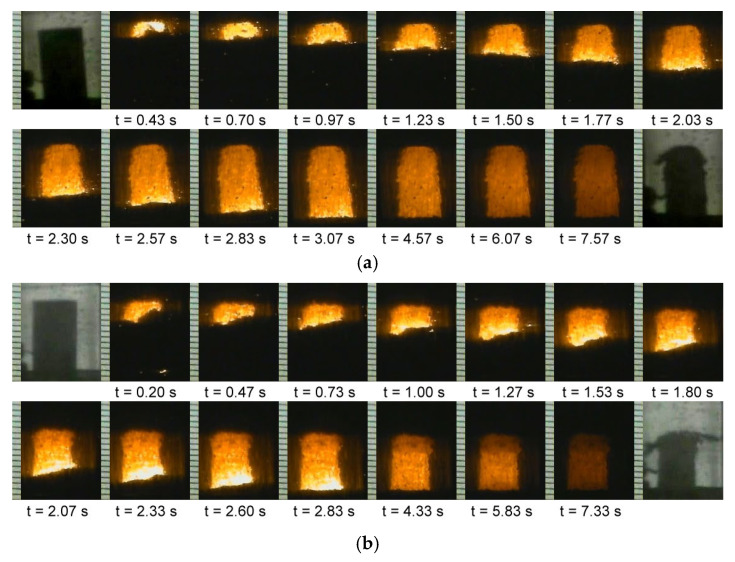
Time sequences of recorded film images illustrating the self-propagating combustion wave traversing the powder compacts with compositional ratios of (**a**) Ti:Al:C = 2:1:1 and (**b**) Ti:Al:C = 2:1.2:0.8.

Figure 1a,b illustrate two sequences of the recorded film images displaying the propagation of the combustion wave along the powder compacts with stoichiometric ratios of Ti:Al:C = 2:1:1 and 2:1.2:0.8, respectively. It is evident that upon ignition, a distinct combustion front formed and traversed the entire sample in a self-sustaining manner. This provides proof that the combustion reaction of this study is exothermic enough to maintain its self-sustainability. In the Ti-Al-C reaction system, the reaction of carbon with Ti to form TiC (with the heat of formation, ΔH_f_ = −184.1 kJ/mol) is much more energetic than the reaction between Ti and Al to produce TiAl and Ti_3_Al (ΔH_f_ = −75.3 and −97.9 kJ/mol) [39,40]. That is, the progression of the combustion wave through the Ti-Al-C powder compact is mainly sustained by the reaction of carbon with Ti.

As reported by Thomas and Bowen [28], the addition of excess Al acting as a diluent caused a decrease in combustion temperature but offered the potential to control the exothermicity of the SHS process. Calculations of the adiabatic temperature (T_ad_) showed that the stoichiometric powder sample of Ti:Al:C = 2:1:1 had T_ad_ of 2368 K, while a lower value of 2190 K was obtained for the Al-excessive sample of Ti:Al:C = 2:1.3:1 [28]. The adiabatic temperature satisfies the criteria proposed for the combustion reaction to be self-sustaining [41,42]. Experimental evidence in this study confirmed self-propagating combustion.

**Figure 2:**
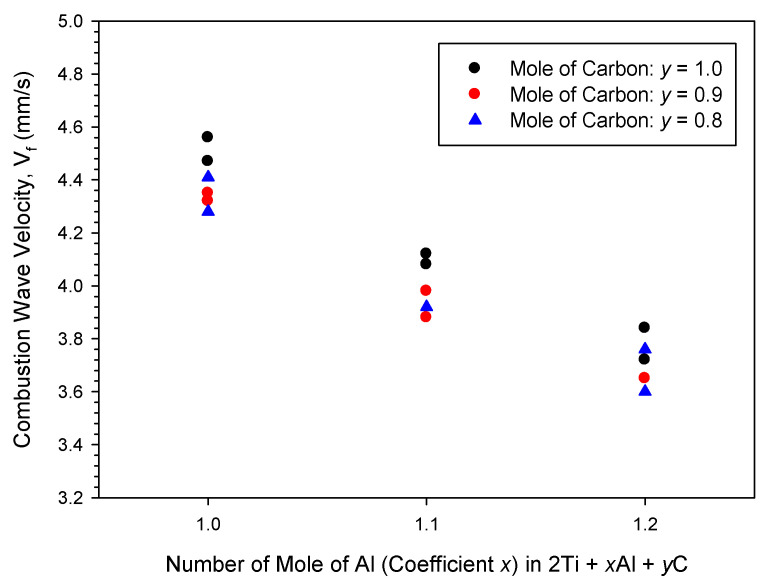
Effects of Al and carbon contents on combustion front velocity in elemental powder compacts with different compositional ratios.

The effects of Al and carbon contents in the reactant mixture on combustion wave velocity are presented in Figure 2. For the powder compacts with stoichiometric carbon of *y* = 1.0, as revealed in Figure 2, the combustion velocity decreased from 4.6 to 3.7 mm/s as the Al content increased from *x* = 1.0 to 1.2. Likewise, the combustion front velocity decreased from 4.3 to 3.6 mm/s for the samples with less carbon at *y* = 0.8. This suggested that excess Al resulted in deceleration of the combustion wave, because of a lower combustion temperature for the Al-excessive sample as reported by Thomas and Bowen [28]. As the content of carbon was reduced from *y* = 1.0 to 0.8, the combustion velocity varied between 4.6 and 4.3 mm/s for the sample of *x* = 1.0, and between 3.8 and 3.6 mm/s for the powder compact of *x* = 1.2. Only a slight decrease in the combustion velocity was observed by reducing carbon content. Namely, the influence of deficient carbon on the combustion velocity was not as pronounced as that of excess Al. This might be because excess Al not only had a cooling impact on combustion but also favored the formation of TiAl instead of Ti_3_Al. Deficient carbon led to the formation of sub-stoichiometric TiC_x′_, which was considered as one of the important intermediates [24,27,32], and its formation exothermicity was almost unaffected by a small deviation in carbon content.

**Figure 3:**
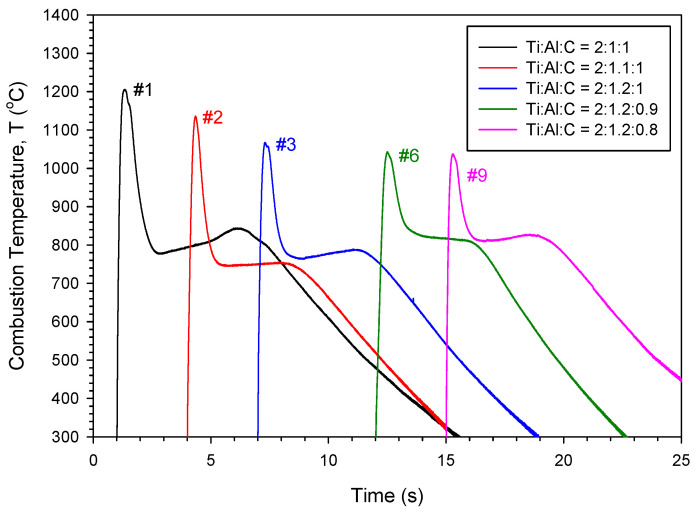
Combustion temperature profiles of elemental powder compacts with different compositional ratios. (The combustion temperature profiles were horizontally shifted along the time axis to avoid overlapping of the curves).

The propagation modes of the combustion wave in the SHS process, such as the planar, spinning, and pulsating modes, are subject to the conduction heat transfer from the reaction zone to unburned section, and hence, the combustion front temperature plays a critical role in the spreading rate of the combustion wave [43,44]. Figure 3 plots five combustion temperature profiles associated with sample compacts of different compositional ratios. The number appearing beside each profile represents the sample number denoted in Table 1. All profiles display an abrupt rise followed immediately by a rapid decline, which is typical of the SHS reaction characterized by a fast combustion wave and a narrow reaction zone. The summit value was regarded as the combustion front temperature (T_c_). After passage of the combustion wave, the decrease in temperature was halted by a nearly plateau period, beyond which a slow decrease in temperature continued. The presence of the plateau region in the contour signifies the occurrence of a volumetric reaction after the combustion front. Since it was not easy for the synthesis reaction to finish inside a rapid and narrow combustion zone, the reaction could continue in a bulk fashion.

As indicated in Figure 3, the combustion front temperature of the samples containing stoichiometric carbon of *y* = 1.0 decreased from 1206 °C (Sample #1) to 1067 °C (Sample #3) as the Al content increased from *x* = 1.0 to 1.2. This confirms the diluent effect of excess Al on combustion. On the other hand, the increase in Al brough about a change in the intermetallic phase formed during the SHS process from Ti_3_Al to TiAl. As mentioned above, the heat of formation of TiAl is less than that of Ti_3_Al [39,40]. Both Ti_3_Al and TiAl are potential intermediate phases to form Ti_2_AlC [20,24,29,31]. The combustion front temperatures of two carbon-deficient samples (#6 and #9) were comparable, reaching about 1040 °C, which was slightly lower than that of Sample #3. This provides proof for the slight influence of carbon content between *y* = 0.8 and 1.0 on the combustion exothermicity. Another important intermediate phase was TiC, which could be in a sub-stoichiometric form TiC_x′_ (0.8 < x′ < 1.0). The formation of sub-stoichiometric TiC_x′_ has been specified in the production of Ti_2_AlC [24,27,32]. Experimental evidence indicated that the variation in combustion front temperature with Al and carbon contents is consistent with that of combustion wave velocity. In addition, the temperature of the plateau region ranged between 760 °C and 820 °C. This plateau period represented the bulk reaction taking place inside the sample rather than on the surface. Because the thermocouple was mounted on the sample surface, the recorded temperature of the plateau region could be lower than the actual reaction temperature.

In general, smaller particles with a larger specific surface area could enhance contact between reactant powders, lower the ignition temperature, increase the combustion wave velocity, and raise the reaction temperature. Moreover, smaller reactant particles could result in finer-grained and more homogeneous product microstructures. The effect of sample density on the reaction temperature and combustion wave velocity has been explored in our previous study [30]. The influence of sample density on the combustion velocity is dependent on two opposing effects. As the sample density increases, the intimate contact between reactant particles is improved and thus enhances the combustion propagation rate. Oppositely, the thermal conductivity of the sample also increases with compaction density, which leads to more conduction heat loss from the reaction zone to the entire sample and hence causes a decrease in the flame spreading speed.

### 3.2. Phase Composition and Microstructure Analyses of Synthesized Products

**Figure 4:**
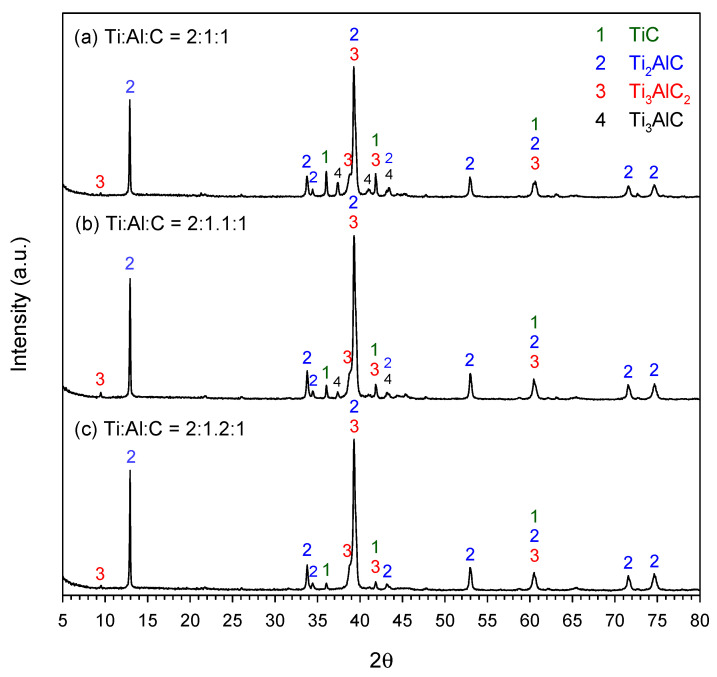
XRD patterns of the end products of elemental powder compacts with compositional ratios of (**a**) Ti:Al:C = 2:1:1, (**b**) Ti:Al:C = 2:1.1:1, and (**c**) Ti:Al:C = 2:1.2:1.

Figure 4a–c present the XRD patterns of synthesized products from elemental powder compacts with compositional ratios of Ti:Al:C = 2:1:1, 2:1.1:1, and 2:1.2:1, respectively. The content of carbon in the samples of Figure 4 was under a stoichiometric amount, i.e., *y* = 1.0. As revealed in Figure 4, three ternary carbides, Ti_3_AlC_2_, Ti_3_AlC, and Ti_2_AlC, and one binary carbide, TiC, were identified in the end products. Ti_2_AlC was the dominant phase. With the addition of excess Al, Ti_3_AlC was found to decrease in Figure 4b (Ti:Al:C = 2:1.1:1) and finally disappeared in Figure 4c (Ti:Al:C = 2:1.2:1). Moreover, excess Al resulted in a decrease in the peak intensity of TiC and Ti_3_AlC_2_. That is, the formation of Ti_2_AlC was enhanced by the introduction of excess Al.

**Figure 5:**
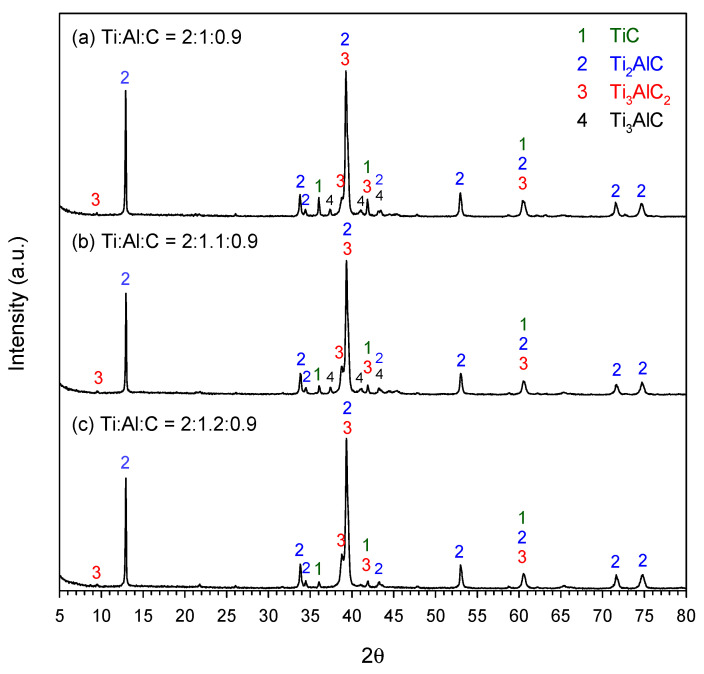
XRD patterns of the end products of elemental powder compacts with compositional ratios of (**a**) Ti:Al:C = 2:1:0.9, (**b**) Ti:Al:C = 2:1.1:0.9, and (**c**) Ti:Al:C = 2:1.2:0.9.

For the samples with carbon content of *y* = 0.9, Figure 5a–c show three XRD patterns associated with different Al contents. As also observed in Figure 4, Ti_2_AlC was the dominant carbide, with Ti_3_AlC_2_, Ti_3_AlC, and TiC as the minor phases. It is evident that minor phases were decreased by the addition of excess Al. A comparison between Figure 4 and Figure 5 indicates lower proportions of Ti_3_AlC_2_, Ti_3_AlC, and TiC existing in the end products of carbon-lean samples.

The weight percentages of Ti_3_AlC_2_, Ti_2_AlC, and TiC in the end products of the powder compacts of Equation (1) were determined using Equations (3)–(5) and are summarized in Table 1. For Sample #1 with an exact stoichiometry of Ti:Al:C = 2:1:1, the final product comprised Ti_2_AlC of 79.5 wt.%, Ti_3_AlC_2_ of 9.8 wt.%, and TiC of 10.7 wt.%. The addition of extra Al in Samples 2 and #3 enhanced the evolution of MAX carbides. It should be noted that a small amount of Ti_3_AlC existed in the products of Samples #1 and #2. An extra amount of Al, by 20 at.% not only increased the yield of Ti_2_AlC but also prevented the formation of Ti_3_AlC. A final product consisting of 84.1 wt.% Ti_2_AlC, 13.0 wt.% Ti_3_AlC_2_, and 2.9 wt.% TiC was obtained from the Al-rich sample of Ti:Al:C = 2:1.2:1 (Sample #3).

To estimate the content of Ti_3_AlC, the diffraction peak of Ti_3_AlC at 2θ = 37.4° was selected, and the peak intensity was integrated. The weight fraction of Ti_3_AlC was estimated from the intensity ratio of the integrated value of the Ti_3_AlC peak to that of Ti_2_AlC at 2θ = 13.0°, since Ti_3_AlC was considered as one of the intermediate phases to form Ti_2_AlC through Equation (10). The estimated weight fractions of Ti_3_AlC are presented in parentheses in the Ti_2_AlC column in Table 1. For example, Table 1 indicates that the weight fraction of Ti_2_AlC is 75.9 wt.% in Sample #1, within which there is about 8.7 wt.% Ti_3_AlC. Except for Sample #1, the other Ti_3_AlC-containing products had low fractions of Ti_3_AlC, of about 2.1–3.6 wt.%.

When compared to the impact of excess Al, the reduction of carbon contributed to greater improvement in the yield of Ti_2_AlC. Table 1 indicates that high yields of Ti_2_AlC, of about 87.2 and 87.5 wt.%, were obtained from the carbon-lean test specimen of Ti:Al:C = 2:1:0.9 and 2:1:0.8 (Samples #4 and #7), respectively. A further increase in the formation of Ti_2_AlC was accomplished from off-stoichiometric samples simultaneously with excess Al and deficient carbon. The highest fraction of Ti_2_AlC was obtained from the sample compact of Ti:Al:C = 2:1.2:0.9 (Sample #6), which produced 91.9 wt.% Ti_2_AlC, 4.2 wt.% Ti_3_AlC_2_, and 3.9 wt.% TiC. For the sample of Ti:Al:C = 2:1.2:0.8 (Sample #9), the resulting product also contained Ti_2_AlC of more than 90 wt.%. Moreover, no trace of Ti_3_AlC was detected in the products of Samples #6 and #9. As listed in Table 1, comparable product compositions were observed for the carbon-lean samples with a carbon reduction of 10 and 20 at.%, i.e., *y* = 0.9 and 0.8. Based upon the experimental results of this study, the elemental sample containing excess Al by 20 at.% and reduced carbon by 10 at.% is recommended for the synthesis of Ti_2_AlC by the SHS route.

Many potential reaction steps could be involved in the formation mechanism of Ti_2_AlC in the Ti-Al-C system via the SHS process. Firstly, the reaction of carbon with Ti producing TiC, as shown in Equation (6), acts as the initiation reaction and is the major heat-releasing step [34], because the SHS process counts on a substantially exothermic reaction to sustain its auto-propagation. Equation (6) features a high reaction heat (ΔH_f_ = −184.1 kJ/mol) and adiabatic temperature (T_ad_ = 3120 K) [34]. By taking advantage of the high exothermicity of Equation (6), the elemental reactions between Ti and Al were triggered and generated Ti_3_Al and TiAl, as expressed in Equations (7) and (8), respectively. Equation (9) indicates that Ti_3_Al could further react with carbon to form Ti_3_AlC [20,24], which explains the presence of Ti_3_AlC in some of the products. Finally, Equations (10) and (11) signify two possible reaction paths for the formation of Ti_2_AlC [20,24]. One represents the reaction between TiAl, Ti_3_AlC, and carbon. The other is the reaction of TiAl with TiC. In addition, according to Equation (12), a certain amount of Ti_2_AlC and TiC could react at high temperatures to form Ti_3_AlC_2_ [20,34].

(6) Ti+C→TiC

(7) $$3Ti+Al\to {Ti}_{3}Al$$

(8) Ti+Al→TiAl

(9) $${Ti}_{3}Al+C\to {Ti}_{3}AlC$$

(10) $$TiAl+{Ti}_{3}AlC+C\to {2Ti}_{2}AlC$$

(11) $$TiAl+TiC\to {Ti}_{2}AlC$$

(12) $${Ti}_{2}AlC+TiC\to {Ti}_{3}AlC_{2}$$

It has been proposed that excess Al could compensate for its evaporation loss during the SHS process, due to the high vapor pressure and low melting temperature of Al [45,46]. Another benefit of excess Al is associated with the formation of intermetallic phases. As indicated in the Ti-Al phase diagram [47,48], the TiAl phase has a wide homogeneity range from 46.7 to 66.5 at.% Al. That is, the Al-rich samples (with an extra amount of 10–20 at.%) of this study facilitated the formation of TiAl rather than Ti_3_Al. Also, the formation Ti_3_AlC could be avoided when the major intermetallic phase was TiAl.

Many studies [24,27,32] pointed out that TiC produced in Equation (6) is in a sub-stoichiometric form, TiC_x′_. The Ti-C phase diagram [49] indicates the homogeneity range of TiC being from 32 to 48.8 at.% carbon, which validates the sub-stoichiometric composition. In this study, the carbon-lean samples with carbon reduced by 10–20 at.% fit within the homogeneity range of TiC and are beneficial to the production of sub-stoichiometric TiC. Once the condition favors the production of TiC, the reaction path to produce Ti_2_AlC could prefer Equation (11) to Equation (10). Therefore, it is proposed that for the elemental sample of Ti:Al:C = 2:1.2:0.9, the formation mechanism of Ti_2_AlC via SHS is governed by a reaction sequence including Equations (6), (8) and (11).

**Figure 6:**
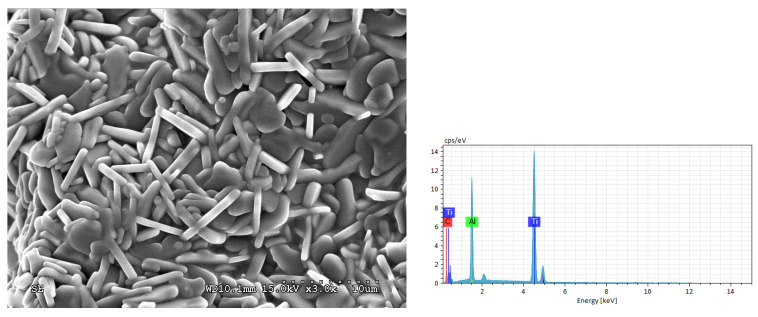
SEM photo and EDS pattern of the product synthesized from an elemental powder compact with Ti:Al:C = 2:1:1.

The SEM photo and EDS pattern presented in Figure 6 exhibit the microstructure of fracture surface and the elemental composition of final product obtained from the powder compact of Ti:Al:C = 2:1:1. The SEM image shown in Figure 6 can be considered as the representative microstructure of the entire sample, since almost all the SEM images exhibited a similar morphology. The EDS spectrum was obtained from the full scan of the SEM image of Figure 6. A laminated microstructure, which is typical of the MAX ternary carbide, is noticeable. The product grains are randomly staggered and feature a smooth surface and a platelet shape. Plate-like grains are 4–8 μm in size and around 1 μm in thickness. An atomic ratio of Ti:Al:C = 50.8:22.7:26.5 was determined from the EDS analysis, which implied a mixture composed of Ti_2_AlC as the dominant phase and Ti_3_AlC_2_ as the minor phase. It has been realized that for EDS, the detection of elements like carbon (C), nitrogen (N), and oxygen (O) is difficult due to low X-ray yields and high absorption. The light element error can be lowered to about 1.4 at.%. The elemental concentrations of Ti_2_AlC products synthesized in this study were 71–73.5 wt.% for Ti, 17.5–18.5 wt.% for Al, and 7.5–8.5 wt.% for carbon. These ranges are within the EDS detection limit, which defines the major element as having content larger than 10 wt.% and the minor element content of 1–10 wt.%.

**Figure 7:**
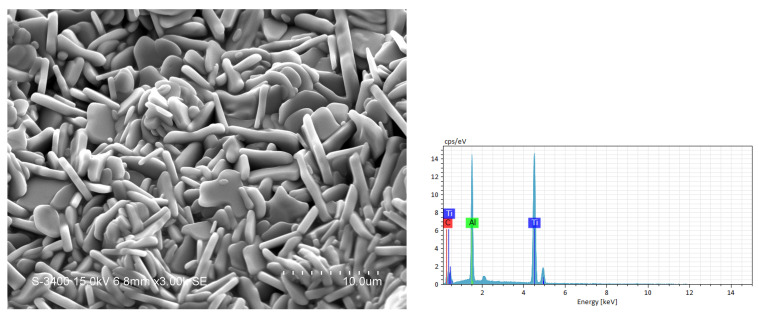
SEM photo and EDS pattern of the product synthesized from an elemental powder compact with Ti:Al:C = 2:1.2:0.9.

Figure 7 displays the SEM and EDS analyses associated with the end product of the sample of Ti:Al:C = 2:1.2:0.9. The morphology of product grains is similar to that observed in Figure 6. Microstructural characteristics of the MAX phase are evident. It should be noted that the characteristic peak of Al has a stronger intensity in Figure 7 than in Figure 6. Since the EDS spectrum was obtained from the full scan of the SEM image in Figure 7, it could represent a higher Al content within this area. The atomic proportion of Ti:Al:C = 51.8:23.8:24.4 was obtained from the EDS analysis of Figure 7. The elemental composition matched well with Ti_2_AlC. This result is consistent with the fact that the product synthesized from the sample of Ti:Al:C = 2:1.2:0.9 contained about 92 wt.% of Ti_2_AlC.

## 4. Conclusions

The MAX carbide Ti_2_AlC was fabricated using elemental Ti-Al-C powder compacts via combustion synthesis in the SHS mode. The initial compositional ratios of Ti:Al:C were varied from 2:1:1 to 2:1.2:0.8 to study the effects of excess Al and deficient carbon on the formation of Ti_2_AlC. Experimental evidence showed that the reaction exothermicity is sufficient to support the self-propagating combustion process. Excess Al by 10–20 at.% decreased the combustion temperature from 1206 °C to 1067 °C and lowered the combustion wave velocity from 4.6 to 3.7 mm/s. This was attributed partly to the dilution effect of Al, and partly to the formation of TiAl as an intermediate instead of Ti_3_Al. For the samples with carbon reduced by 10–20 at.%, however, the combustion temperature and velocity were relatively little affected. This was probably due to the fact that another intermediate TiC was generated under a sub-stoichiometric form.

From the sample with an exact stoichiometry of Ti:Al:C = 2:1:1, the as-synthesized product consisted of 79.5 wt.% Ti_2_AlC, 9.8 wt. Ti_3_AlC_2_, 10.7 wt.% TiC, and a small amount of Ti_3_AlC. The addition of excess Al by 20 at.% (Ti:Al:C = 2:1.2:1) increased the yield of Ti_2_AlC to about 84 wt.% and avoided the formation of Ti_3_AlC. A decrease in carbon by 10 and 20 at.% (Ti:Al:C = 2:1:0.9 and 2:1:0.8) contributed to greater improvement in the formation of Ti_2_AlC, and a Ti_2_AlC fraction of about 87 wt.% was obtained. In this study, the final product with the highest content of Ti_2_AlC was synthesized from the test specimen of Ti:Al:C = 2:1.2:0.9, which represents an off-stoichiometric composition together with excess Al and deficient carbon. The optimum phase composition was 91.9 wt.% Ti_2_AlC, 4.2 wt.% Ti_3_AlC_2_, and 3.9 wt.% TiC. Because excess Al facilitated the formation of TiAl, and deficient carbon favored the production of sub-stoichiometric TiC, the formation of Ti_2_AlC from the combination reaction between TiC and TiAl was enhanced. The as-synthesized Ti_2_AlC grains were in a platelet shape and were stacked tightly into a laminated microstructure, which is typical of the MAX carbide.

## Figures and Tables

**Table 1:** Weight percentages of Ti_3_AlC_2_, Ti_2_AlC, and TiC in the end products of sample compacts of Equation (1) with different compositions.

Sample No.	Ti:Al:C	Weight Percentage (wt.%)		
		Ti_2_AlC	Ti_3_AlC_2_	TiC
1 *	2:1:1	79.5 (8.7)	9.8	10.7
2 *	2:1.1:1	82.1 (3.3)	13.8	4.1
3	2:1.2:1	84.1	13.0	2.9
4 *	2:1:0.9	87.2 (3.5)	5.6	7.2
5 *	2:1.1:0.9	88.7 (2.2)	6.8	4.5
6	2:1.2:0.9	91.9	4.2	3.9
7 *	2:1:0.8	87.5 (3.6)	3.1	9.4
8 *	2:1.1:0.8	88.5 (2.1)	7.8	3.7
9	2:1.2:0.8	91.0	6.1	2.9

* In addition to Ti_2_AlC, Ti_3_AlC_2_, and TiC, a small amount of Ti_3_AlC was present in the product. The values listed in parentheses in the Ti_2_AlC column represent the weight fractions of Ti_3_AlC.

## Data Availability

The original contributions presented in this study are included in the article. Further inquiries can be directed to the corresponding author.
